# Supplementary material for: An open protocol for modeling T Cell Clonotype repertoires using TCRβ CDR3 sequences
Source: BMC Genomics. 2023 Jun 26;24:349. doi: 10.1186/s12864-023-09424-z (PMC10291816; doi:10.1186/s12864-023-09424-z)
Supplement: Supplementary file 1 — Additional file 1: Supplementary Figure 1. ST Count Distribution in presence of DNA. Supplementary Figure 2. Normalization reduces spread in presence of DNA. Supplementary Figure 3. Concordance analysis of T cell repertoire metrics. Supplementary Figure 4. Competition between gDNA and ST during TCR sequencing. Supplementary Figure 5. Reproducibility analysis: Drop-outs are frequent even for the top clones. Supplementary Table 1. Primer Sequences. Supplementary Figure 6. TCR sequencing pipeline schema. Supplementary Figure 7. Monoclonal amplification check. [file 12864_2023_9424_MOESM1_ESM.docx]

**Supplementary Figure 1. ST Count Distribution in presence of DNA.**


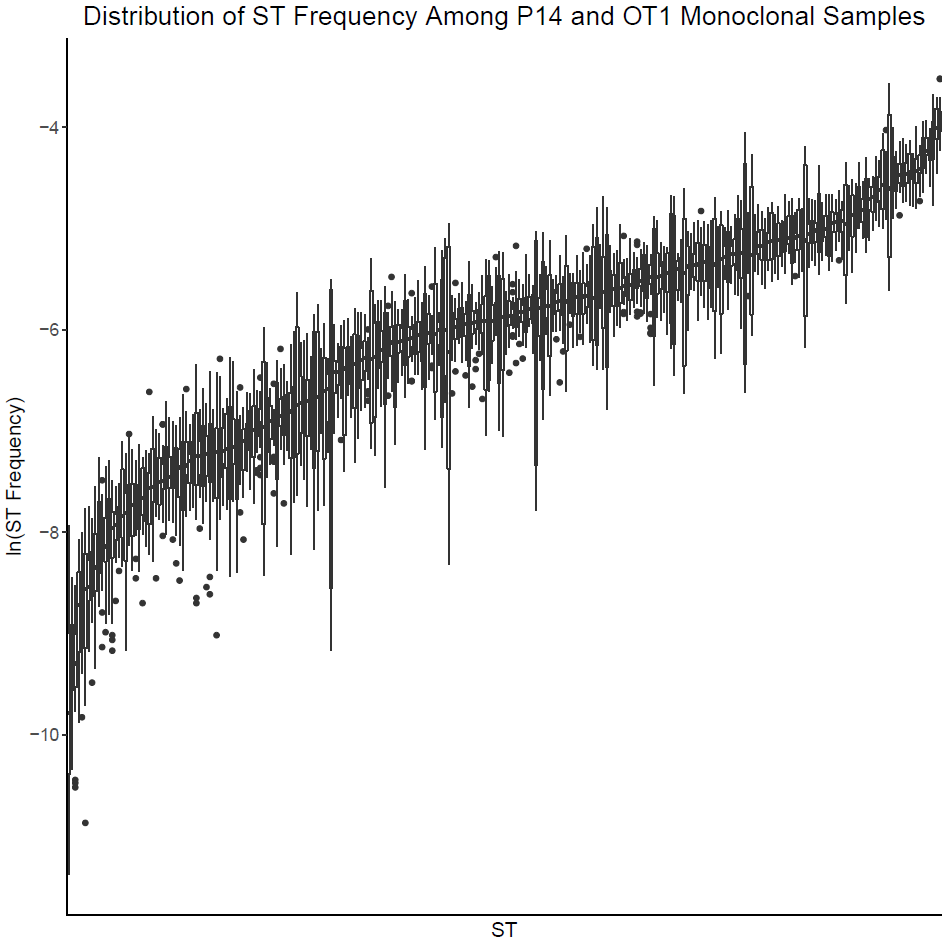


ST counts were obtained from samples described in the *Transgenic TCR data sets,* where 24 samples of gDNA from P14 and OT1 TCR transgenic mice were processed along with an equimolar mixture of ST. OT1 was amplified by the primer pair (V12-1,2, J2.7), and P14 was amplified by the primer pair (V13-3, J2-4). As with the ST-only samples, when TCR clonotypes were present in the samples along with the ST, the observed variation in the ST counts was caused by the amplification biases of the different primer pairs, rather than by sample to sample variation.


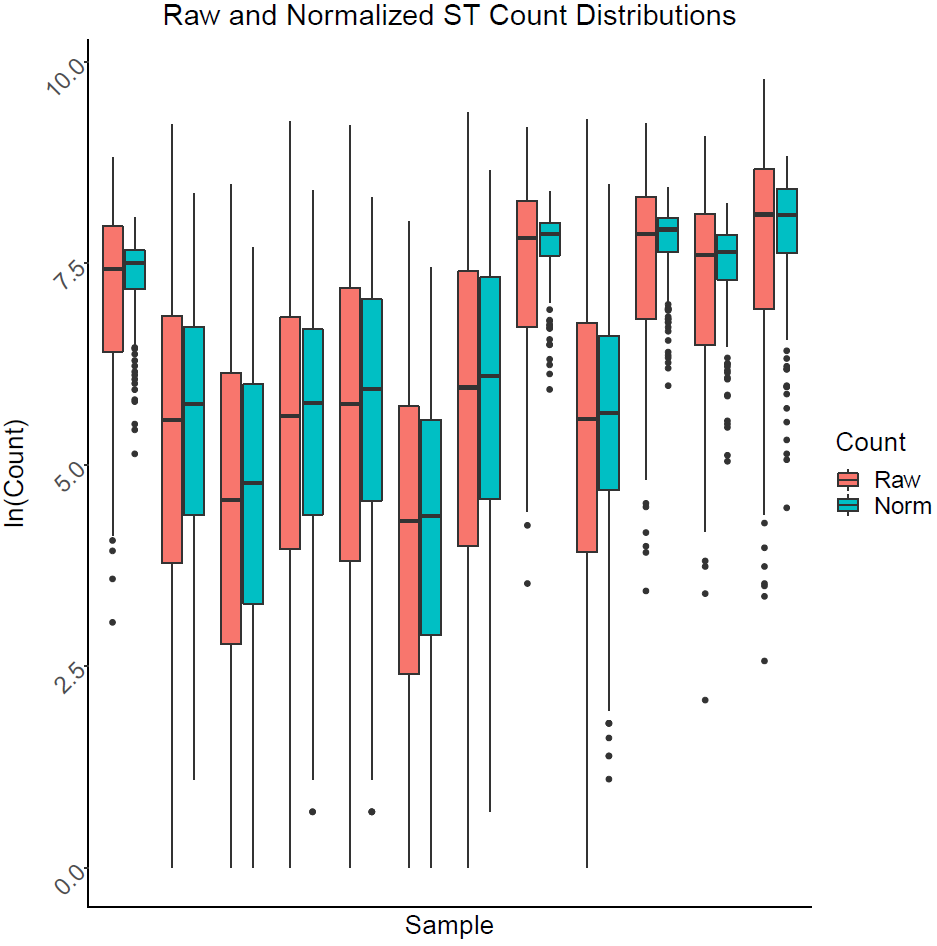


**Supplementary Figure 2. Normalization reduces spread in presence of DNA.**

gDNA from 12 murine mesothelioma specimens were amplified along with ST (described in the Data Sets section) where the reduction in ST count spread in the presence of DNA before (red) and after (cyan) normalization was plotted.

**Supplementary Figure 3. Concordance analysis of T cell repertoire metrics.**


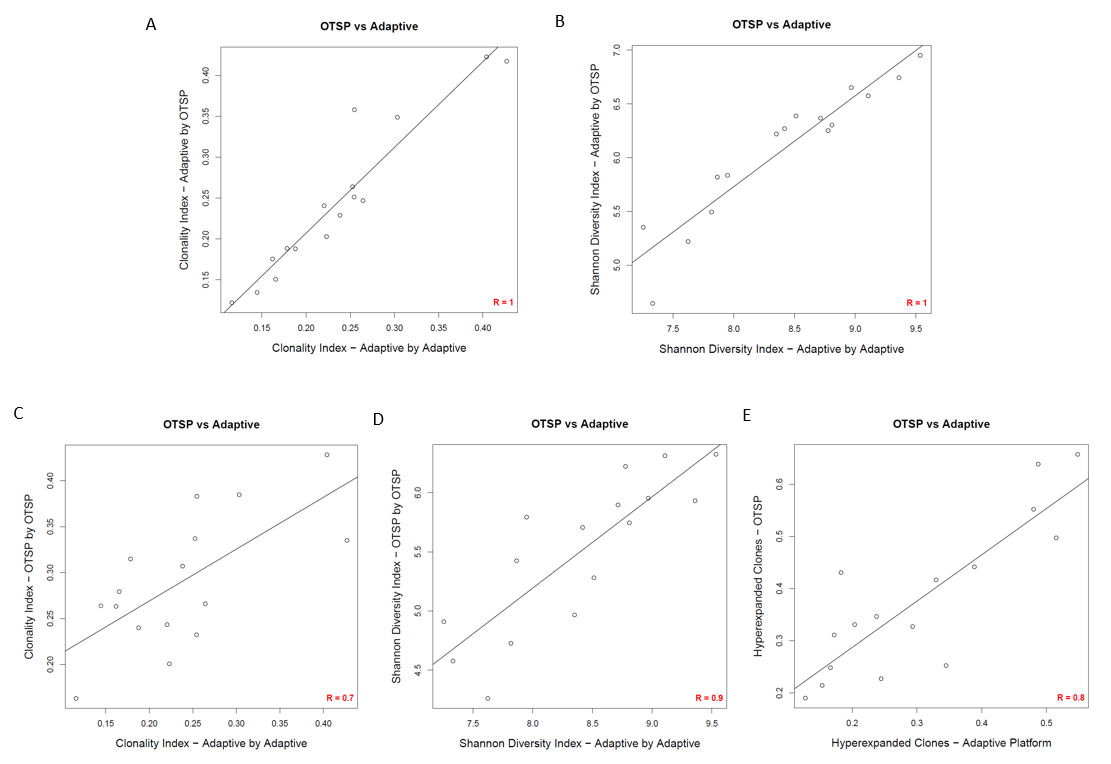


Concordance analysis comparing commercial and in-house pipelines. gDNA from PDAC tumor samples were evaluated based on a commercial platform (Adaptive Biotech.) where sequences were compared based on output from Adaptive Biotechnology versus OTSP for Clonal index (R=1) and Shannon diversity index (R=1) **(A-B),** concordance between the Adaptive Biotech platform versus OTSP for Clonal index (R=0.7) and Shannon diversity index (R=0.9) **(C-D),** and concordance between the two pipelines for the frequency of hyperexpanded clones (R=0.8) **(E).** p<0.001 for all comparisons with Spearman correlation analysis. Data derived from samples described in the *Byrne et al. data set.*

**Supplementary Figure 4. Competition between gDNA and ST during TCR sequencing.**


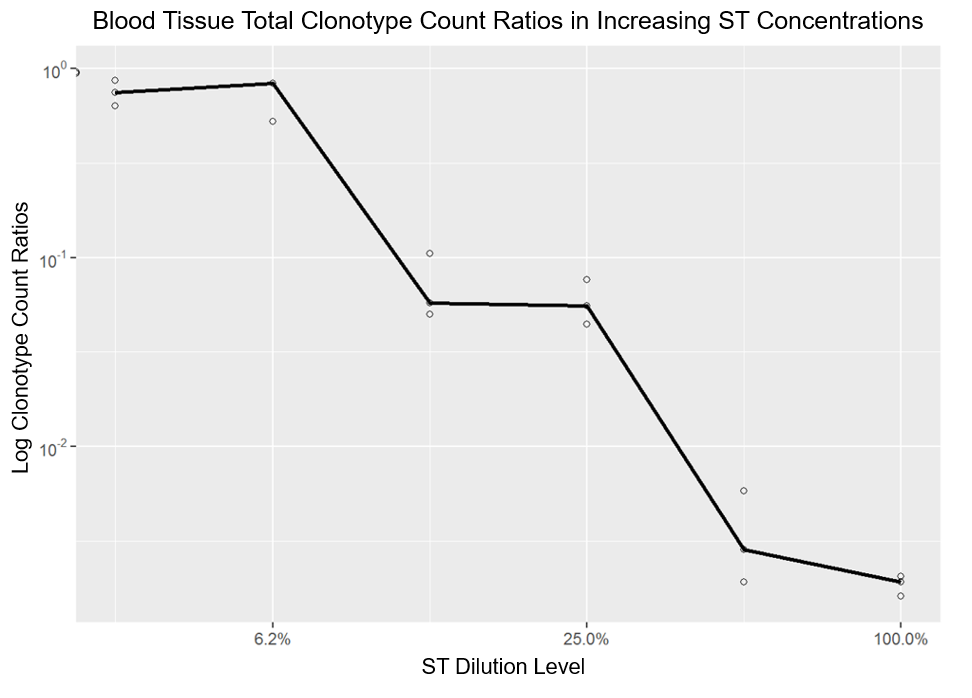


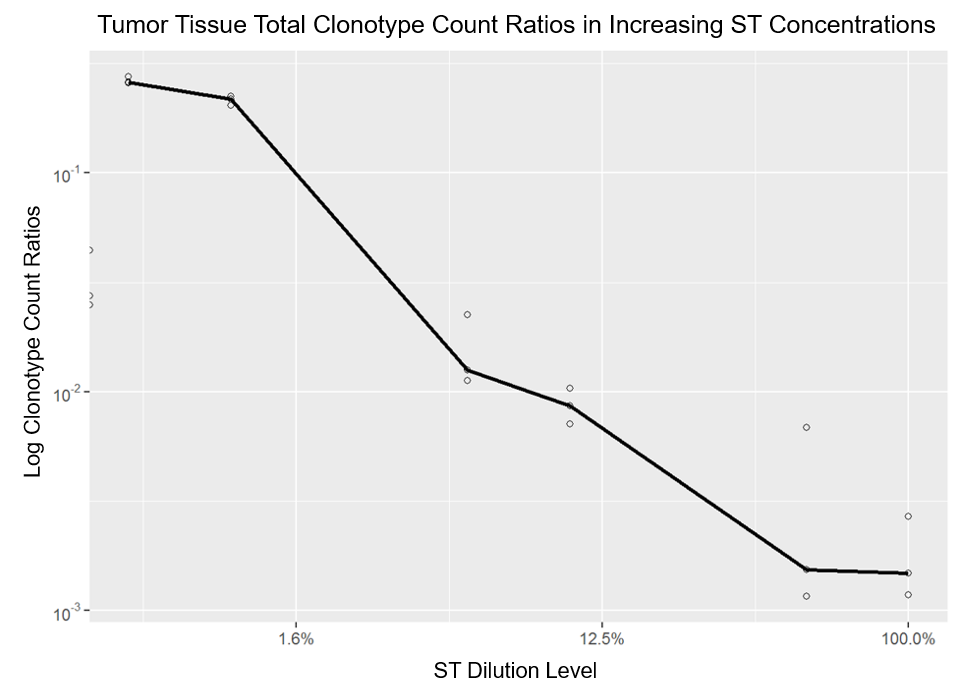


Three replicates of 600 ng of gDNA isolated from peripheral blood leukocytes was added to all levels of dilutions (described in the Data Sets section) and for no ST samples for a total of 21 samples plus three replicates of 600 ng of mouse mesothelioma tumor DNA were added to all levels of dilutions and for no ST samples for a total of 21 samples. When the gDNA amount was kept constant at 600 ng, the increasing (relative) concentration of ST lead to decreasing detectability of clonotypes for both type of tissues, showing the competition between DNA and ST occurring during TCR sequencing.

**Supplementary Figure 5. Reproducibility analysis: Drop-outs are frequent even for the top clones.**


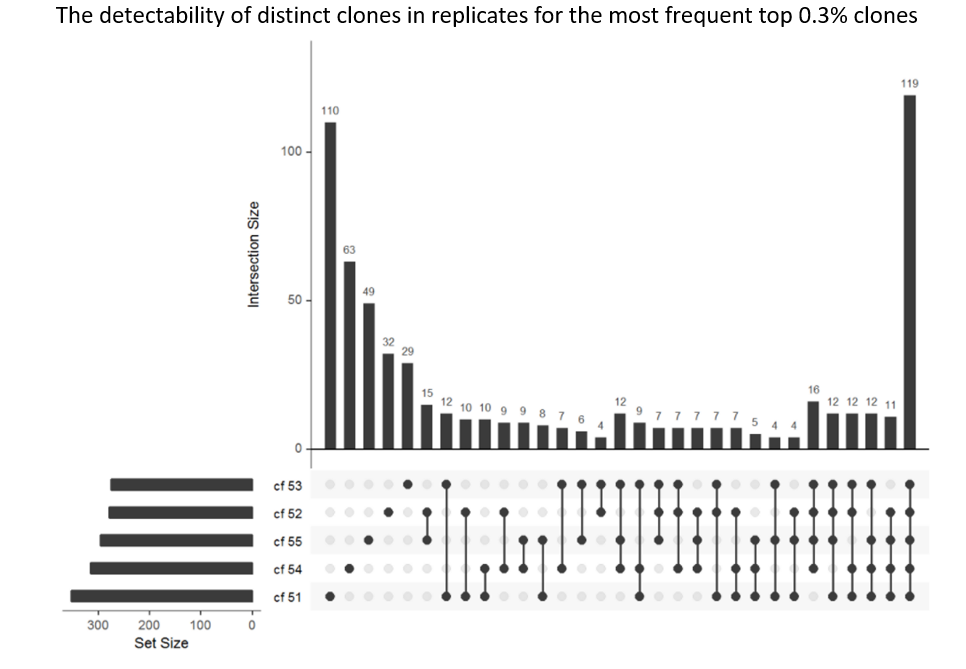


The detectability of distinct clones in five replicates for the most frequent 0.3% clonotypes from wild type spleen tissue is shown. (Data derived from samples described in the *WT spleen data set*.) Vertical bars indicate the frequency of distinct clones detected in replicates, where the number on top of the vertical bars indicates the total number of distinct clones detected in the replicates. On the bottom left, the five replicates (samples cf51-cf55) and the number of distinct clones detected in each replicate is represented by horizontal bars with set size scale. The round dots are black if a particular clone was detected in the corresponding replicate shown at the very left. The connected black dots indicate how many and in which replicates distinct clones were detected out of five replicates. For example, going from right to left, only 119 distinct clonotype were detected in all 5 replicates, 63 clonotypes were detected in 4 samples, 69 in 3 and 90 in 2, respectively. These drop-outs come from the stochastic nature of the sampling.

**
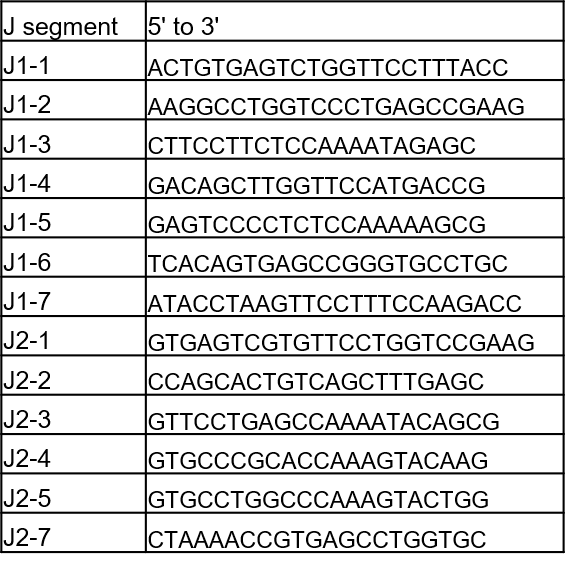

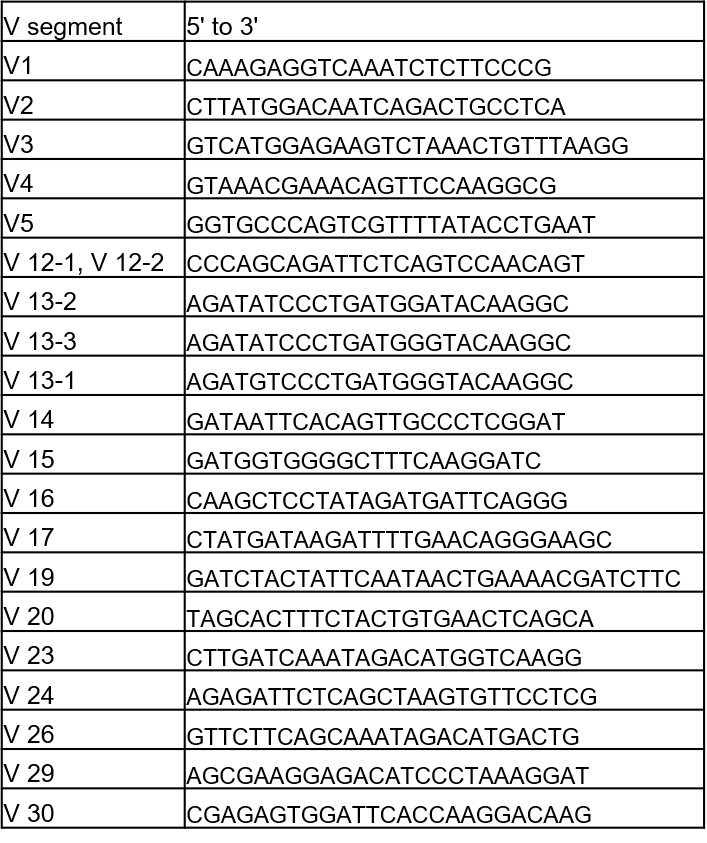
Supplementary Table 1 Primer Sequences.**

B

A

50µm

50µm

50µm

**Supplementary Figure 6. TCR sequencing pipeline schema**


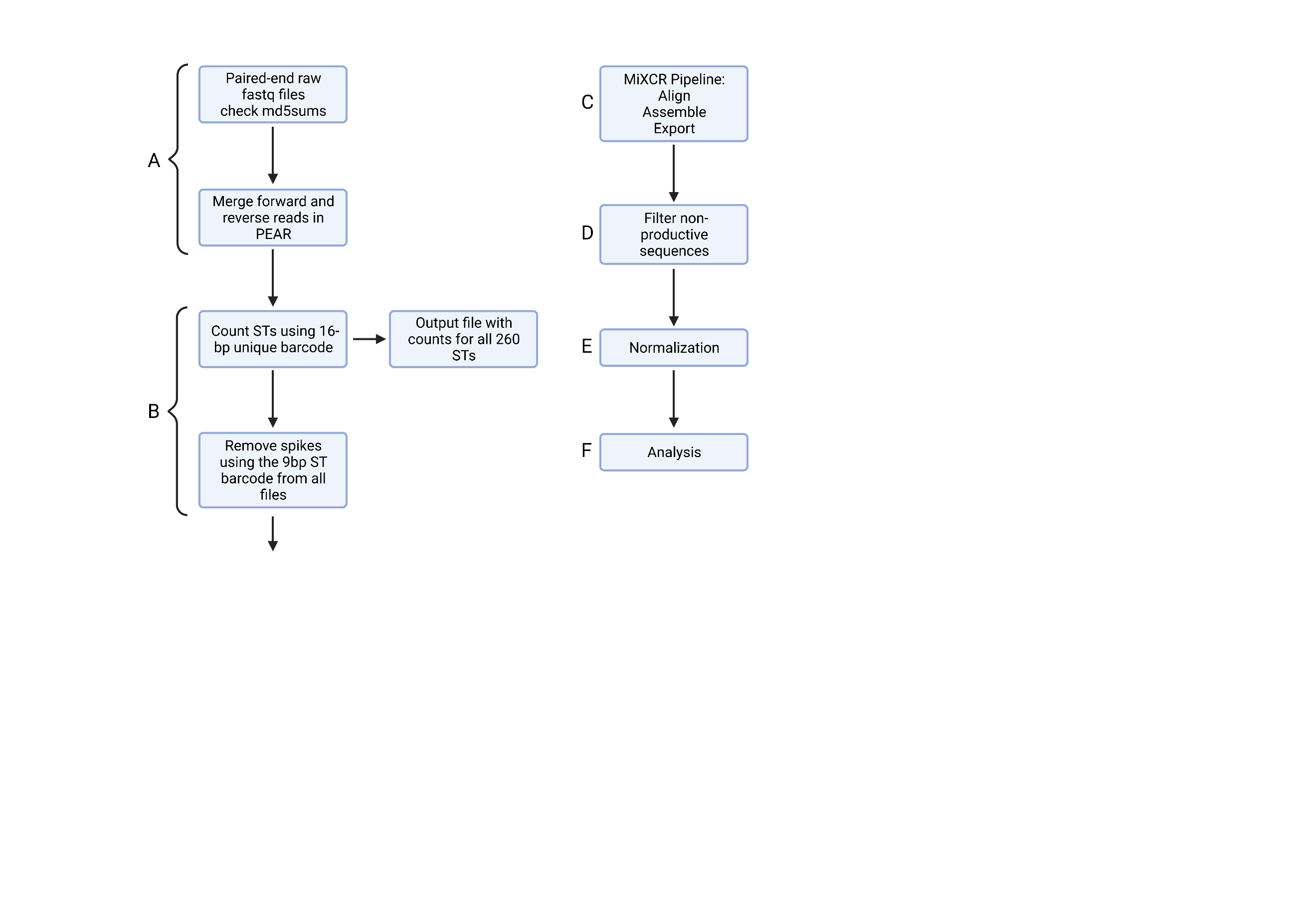


Depiction of TCR sequencing pipeline constructed from both extant software tools (configured for use within the pipeline), and dedicated programs written in-house. Multiple samples are processed in parallel, and quality-control checks provide visibility into the pipeline’s operation. Computation is performed on the 5000 cores using ExaCloud computing cluster. Steps in the pipeline include: **A**. Verification of file integrity and merging of paired-end reads; **B**. ST reads are then identified, quantified, and removed; **C**. Clonotypes are then aligned to reference segments, clustered, and quantified; **D**. Clonotypes containing frameshifts and stop codons flagged, and output converted for use by visualization and analysis software **E**. Clonotype frequencies are then adjusted to account for PCR amplification; **F**. Analytic metrics computed (diversity, clonal expansion and other as applicable) using various tools indicated within the text. Figure created with BioRender.com

**Supplementary Figure 7. Monoclonal amplification check.**

Appropriate amplification and identification of clonal TCR segments was verified using OT1 and P14 monoclonal samples, where OT1 was amplified by the primer pair (V12-1,2, J2.7), and P14 amplified by the primer pair (V13-3, J2-4). One example of each monoclonal sample for appropriate amplification is shown.
