# Supplementary material for: An open protocol for modeling T Cell Clonotype repertoires using TCRβ CDR3 sequences
Source: BMC Genomics. 2023 Jun 26;24:349. doi: 10.1186/s12864-023-09424-z (PMC10291816; doi:10.1186/s12864-023-09424-z)
Supplement: Supplementary file 2 — Additional file 2. [file 12864_2023_9424_MOESM2_ESM.pdf]

Supplementary Appendix 1

Suppose that we have three equimolar spike-in only datasets with possibly different concentrations consisting of 20, 10 and 10 replicate libraries, respectively. Denote their ST counts by $\{C_{ij}^{(k)}: i = 1, \dots 260, j = 1, \dots, n^{(k)}\}$, where $i = 1, \dots 260$ labels ST, $j = 1, \dots, n^{(k)}$ labels replicate libraries within datasets, $k = 1, 2, 3$, and $n^{(1)} = 20$, $n^{(2)} = n^{(3)} = 10$.
Our basic assumption is that for any given ST (i.e. primer-pair) the expected values of the counts for are essentially the same, i.e. that we have

$$\boldsymbol{E}\left(C_{ij}^{(k)}\right) = c^{(k)} m_i, i = 1, \dots, 260, j = 1, \dots, n^{(k)}, k = 1,2,3,$$

up to the concentrations $c^{(1)}, c^{(2)}$, and $c^{(3)}$. Within dataset *k*, natural unbiased estimates of the $c^{(k)} m_i$ are the averages $C_{i\bullet}^{(k)} = \left(n^{(k)}\right)^{-1} C_{i+}^{(k)}$, where $C_{i+}^{(k)} = \sum_{j=1}^{n^{(k)}} C_{ij}^{(k)}$.
These are maximum likelihood estimates (MLE) under the assumption that all the counts are mutually independent Poisson or Negative Binomial random variables with a common overdispersion parameter for each ST. Our goal here is to show how to combine the three estimates of m_i for any given i taking into account the possibly different concentrations. Without loss of generality we can take $c^{(1)} = 1$, and we will write $c^{(2)} = c$ and $c^{(3)} = d$. Here are two approaches to combining the estimates.

Assuming independent Poisson or Negative Binomial distributions. In this case it is a straightforward calculation to show that the MLE of μ_i based on all the counts is

$$\widehat{m}_i = (n^*)^{-1} C_{i+}^{(+)} \text{ where } n^* = 20 + 10\frac{C_{++}^{(2)}}{C_{++}^{(1)}} + 10\frac{C_{++}^{(3)}}{C_{++}^{(1)}}.$$

This makes sense. We sum *all* the counts observed for ST i and divide that by the sum of the effective number of replicates in each dataset, relative to the concentration for dataset 1.

Avoiding strong independence and distributional assumptions. Here we begin by noting that $log\boldsymbol{E}\left(C_{i\bullet}^{(k)}\right) = log\ c^{(k)} + log\ m_i,\ i = 1, \dots, 260, k = 1,2,3$ and make our goal the linear combination of the three approximately unbiased estimates of $\mu_i = log\ m_i$, namely the quantities $l_i^{(k)} = logC_{i\bullet}^{(k)}$, $k = 1,2,3$, correcting for the two offsets $\gamma = log\ c$ and $\delta = log\ d$ of the second and third datasets relative to the first, and taking into account the fact that the first dataset has twice their number of observations. A straightforward weighted least squares estimation process leads to the combined estimate of μ_i as

$$\tilde{\mu}_i = \frac{1}{4}[2l_i^{(1)} + \left(l_i^{(2)} - \tilde{\gamma}\right) + (l_i^{(3)} - \tilde{\delta})]$$

where $\tilde{\gamma} = \frac{1}{260}(l_+^{(2)} - l_{i+}^{(1)})$ and $\tilde{\delta} = \frac{1}{260}(l_i^{(3)} - l_i^{(1)})$. Once we have a combined estimate of $\mu_i = log\ m_i$, we antilog to obtain our estimate $\widetilde{m}_i$ of m_i.

Although the individual ST counts were plausibly negative binomial, they seemed far from independent. As a result, we used the second method to combine the three

sets of estimated count means. Recall that in practice, all we need are the estimates of ratios $m_i/m_\bullet$, so that the concentration terms cancel.
